# The utilization of an ultrasound-guided 8-gauge vacuum-assisted breast biopsy system as an innovative approach to accomplishing complete eradication of multiple bilateral breast fibroadenomas

**DOI:** 10.1186/1477-7819-5-124

**Published:** 2007-10-29

**Authors:** Stephen P Povoski

**Affiliations:** 1 Division of Surgical Oncology, Department of Surgery, Arthur G. James Cancer Hospital and Richard J. Solove Research Institute and Comprehensive Cancer Center, The Ohio State University, Columbus, Ohio, USA

## Abstract

**Background:**

Ultrasound-guided vacuum-assisted breast biopsy technology is extremely useful for diagnostic biopsy of suspicious breast lesions and for attempted complete excision of appropriately selected presumed benign breast lesions.

**Case presentation:**

A female patient presented with 16 breast lesions (eight within each breast), documented on ultrasound and all presumed to be fibroadenomas. Over a ten and one-half month period of time, 14 of these 16 breast lesions were removed under ultrasound guidance during a total of 11 separate 8-gauge Mammotome^® ^excision procedures performed during seven separate sessions. Additionally, two of these 16 breast lesions were removed by open surgical excision. A histopathologic diagnosis of fibroadenoma and/or fibroadenomatous changes was confirmed at all lesion excision sites. Interval follow-up ultrasound imaging revealed no evidence of a residual lesion at the site of any of the 16 original breast lesions.

**Conclusion:**

This report describes an innovative approach of utilizing ultrasound-guided 8-gauge vacuum-assisted breast biopsy technology for assisting in achieving complete eradication of multiple bilateral fibroadenomas in a patient who presented with 16 documented breast lesions. As such, this innovative approach is highly recommended in similar appropriately selected patients.

## Background

The Mammotome^® ^breast biopsy system (Ethicon Endo-Surgery, Inc., Cincinnati, Ohio) is an innovative vacuum-assisted breast biopsy technology which currently has stereotactic, ultrasound, and magnetic resonance imaging (MRI) applications and is available in 8-gauge, 11-gauge, and 14-gauge platforms [1].

Ultrasound-guided applications of the 8-gauge platform of this particular system are well described in the literature [2-10]. In a recently published series of 304 cases, this specific ultrasound-guided 8-gauge vacuum-assisted breast biopsy system was comprehensively evaluated both for the diagnostic biopsy of suspicious breast lesions and for attempted complete excision of appropriately selected presumed benign breast lesions [10]. In this recently reported series, 100% of all suspicious ultrasound lesions were accurately diagnosed (with the demonstration of no false-negative results) and 89% of ultrasound lesions that were presumed to be completely excised by the 8-gauge technique were verified as absent on interval follow-up ultrasound at a median time of 6 months [10].

The present report describes an innovative approach of utilizing an ultrasound-guided vacuum-assisted excision technique with the 8-gauge breast biopsy system described above [2-10] for assisting in achieving complete eradication of multiple bilateral fibroadenomas in a patient who presented with 16 documented breast lesions.

## Case presentation of technical innovation

A 21 year-old, healthy, white female presented to our comprehensive breast health services facility with multiple bilateral breast lesions that were presumed to represent multiple bilateral fibroadenomas. She reported having previously undergone a surgical excision procedure of a fibroadenoma of each of her breasts at a local community hospital at a time of approximately three years prior to her current presentation.

Prior to her current presentation at our facility, the patient was evaluated at an out-of-state university medical center and was noted to have multiple ultrasound lesions within both of her breasts. Due to the multiplicity of the bilateral breast lesions and the complexity of her particular case, the breast surgeon at that out-of-state university medical facility told the patient and her family that her "only realistic option" was for bilateral nipple-sparing mastectomies and expander-to-implant reconstruction.

Upon her presentation to our facility, the patient reported that she had noticed an enlarging mass of her central right breast over the last two years and two smaller palpable masses of her left breast. On clinical breast examination, she had medium-sized breasts (36-C cup size). She had an old surgical scar extending from the 8 to 10 o'clock axis of the periareolar aspect of her right breast and similar surgical scar extending from the 10 to 12 o'clock axis of the periareolar aspect of her left breast. Most remarkably, she was noted to have a 10 cm × 7 cm × 5 cm clinically palpable mass encompassing her entire right breast and an overlying 1.5 cm clinically palpable mass in the in the 3 o'clock axis of the periareolar aspect of her right breast. Two palpable masses were also noted in her left breast, clinically measuring 3 cm in size in the 3 to 4 o'clock axis of the mid-breast field and 2 cm in size in the subareolar region. No axillary, supraclavicular, or cervical adenopathy was noted. The patient also reported being on hormonal contraception for the past five years. For the last 18 months, she was maintained on a combination transdermal contraception product containing norelgestromin (6 mg) and ethinyl estradiol (0.75 mg).

Bilateral breast imaging was performed with both bilateral whole-breast ultrasound and bilateral breast MRI. Ultimately, a total of 16 separate breast lesions were identified on both ultrasound and MRI, consisting of eight lesions documented in each breast (Table 1).

**Table 1:** Characteristics of all sixteen breast lesions visualized on ultrasound examination.

Lesion#	Breast	Ultrasound location† *	Palpable	Ultrasound lesion size (cm)	Ultrasound lesion volume (cm3)‡
1	Right	Central/Subareolar	Yes	9.63 × 8.28 × 4.94	206.24
2	Right	3 o'clock (zone 1)	Yes	1.53 × 1.51 × 1.17	1.41
3	Right	9 o'clock (zone 3)	No	1.25 × 0.76 × 0.40	0.2
4	Right	9 o'clock (zone 3)	No	0.73 × 0.72 × 0.64	0.18
5	Right	9 o'clock (zone 3)	No	0.62 × 0.46 × 0.32	0.05
6	Right	10 o'clock (zone 2)	No	0.66 × 0.64 × 0.41	0.09
7	Right	10 o'clock (zone 3)	No	1.43 × 1.34 × 0.80	0.8
8	Right	11 o'clock (zone 2)	No	1.24 × 0.81 × 0.46	0.24
9	Left	Central/Subareolar	Yes	2.87 × 2.46 × 1.27¶	4.69¶
10	Left	12 o'clock (zone 2)	No	0.96 × 0.77 × 0.36	0.14
11	Left	2 o'clock (zone 2)	No	0.96 × 0.84 × 0.53	0.22
12	Left	2 o'clock (zone 2)	No	0.91 × 0.84 × 0.39	0.16
13	Left	3 to 4 o'clock (zone 1)	Yes	1.39 × 1.32 × 0.77§	0.74§
14	Left	3 to 4 o'clock (zone 2)	Yes	3.30 × 2.85 × 1.48*	7.29*
15	Left	4 o'clock (zone 2)	No	0.87 × 0.79 × 0.36	0.13
16	Left	7 to 8 o'clock (zone 1)	No	0.55 × 0.53 × 0.25	0.04

^† ^The location of each breast lesion seen on ultrasound was reported using its "o'clock" location and its 1, 2, or 3 "zone" location relative to the distance from the nipple (i.e., zone 1 is the central-third of the breast field, zone 2 is the middle-third of the breast field, and zone 3 is peripheral-third of the breast field) [20, 21].

* All sixteen breast lesions visualized on ultrasound were also identified on MRI. However, the three closely approximated lesions (lesion #3, #4, and #5) that were ultimately identified on ultrasound in the 9 o'clock axis of zone 3 of the right breast could not be distinguished as three separate areas of enhancement on MRI, but appeared as one vaguely defined area of enhancement on MRI.

^‡ ^The volume of the ultrasound lesion was calculated using the formula of the volume of an ellipsoid [4/3·π·length axis radius·width axis radius·height axis radius], rather than using the formula of the volume of a cuboid [length·width·height], since the three-dimensional shape of any given ultrasound lesion generally better approximated that of an ellipsoid rather than that of a cuboid [10].

^¶ ^Seven months after stopping hormonal contraception, lesion #9 measured 2.35 × 1.63 × 0.79 cm in size (volume 1.58 cm^3^), consisting of a 166% volume reduction.

^§ ^Four months after stopping hormonal contraception, lesion #13 measured 1.34 × 1.27 × 0.69 cm in size (volume 0.61 cm^3^), consisting of a 37% volume reduction.

^£^Five and one-half months after stopping hormonal contraception, lesion #14 measured 2.12 × 1.91 × 0.98 cm in size (volume 2.08 cm^3^), consisting of a 186% volume reduction.

As a result of the radical and body-altering surgical intervention recommendations for bilateral nipple-sparing mastectomies and expander-to-implant reconstruction that were made by the breast surgeon at an out-of-state university medical center, as well as due to her enlarging bilateral breast masses, both the patient and her parents were highly anxious and concerned. Both the patient and her parents desperately desired an alternative therapeutic approach to deal with her multiple bilateral breast lesions and that could accomplish the same goal of complete removal of all detectable lesions while drastically minimizing the degree of surgical invasiveness and maximizing the cosmetic outcome.

**Figure 1:**
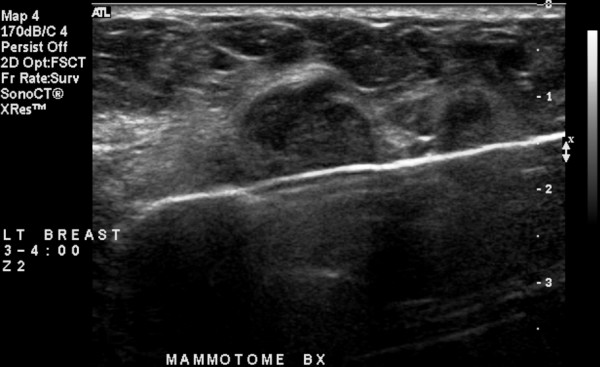
Ultrasound image in longitudinal axis showing the 8-gauge vacuum-assisted device positioned just beneath the ultrasound lesion once the cutter blade has been fully activated across the aperture of the tissue collection chamber.

**Figure 2:**
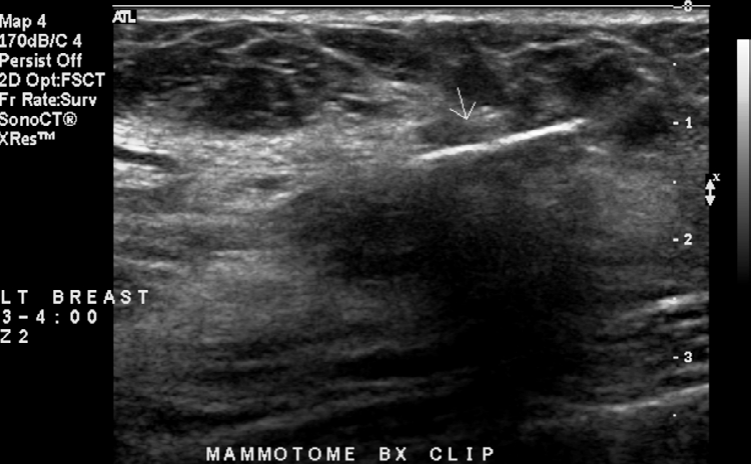
Ultrasound image in longitudinal axis showing a 14-gauge Cormark™ microclip marking the area of the 8-gauge vacuum-assisted excision of the previous ultrasound lesion.

A total of 11 separate 8-gauge vacuum-assisted excision procedures, using the previously described breast biopsy system [2-10], were performed to a total of 14 different ultrasound lesions during seven separate sessions over a ten and one-half month period of time (Table 2). The technique used for all 11 of the ultrasound-guided 8-gauge vacuum-assisted excision procedures performed in this current report has been previously described and reported by the present author [10]. Real-time ultrasound guidance was performed using the Philips HDI 5000 SonoCT system (Philips Medical Systems Andover, Massachusetts), with a variable frequency transducer L12-5 (range 4.75 to 10.0 MHz). After local anesthetic was administered to the proposed 8-gauge vacuum-assisted excision site, a 5 mm skin incision was made with a #11 blade. Then, under real-time ultrasound guidance, the 8-gauge vacuum-assisted device was passed through the 5 mm skin incision and positioned just beneath the ultrasound lesion (Figure 1) and multiple 8-gauge cores were consecutively harvested while sequentially rotating the 8-gauge vacuum-assisted device over an array spanning approximately 180 degrees. Ongoing ultrasound assessment of the progression of lesion excision and final verification of presumed complete lesion excision was performed in real-time in both the longitudinal and transverse planes to the long axis of the 8-gauge device. After completion of core acquisition, the 8-gauge device was removed and a 14-gauge Cormark™ rigid microclip device (Ethicon Endo-Surgery, Inc., Cincinnati, Ohio) was inserted through the same breast parenchymal track under real-time ultrasound guidance for placement of a microclip into the excision cavity (Figure 2). A single microclip was placed at the site of each ultrasound-guided 8-gauge vacuum-assisted excision procedures, except for that of the ultrasound-guided 8-gauge vacuum-assisted excision procedure for lesions 11 and 12, in which case two adjacent microclips were placed. Thereafter, as a standard and to assure adequate hemostasis, manual compression to the breast was performed for approximately ten minutes. No peri-procedural bleeding complications occurred. Finally, the 5 mm incision site was reapproximated with standard adhesive skin closure strips.

**Table 2:** Summary of all eleven separate ultrasound-guided 8-gauge vacuum-assisted excision procedures.

Procedure number	Session number	Lesion excised from Table 1	Duration of time from presentation to procedure in months	Number of 8-gauge cores harvested	Overall estimated 3-dimensions of 8-gauge cores harvested in centimetres (cm)
1	1	10	1.0	10	3.6 × 2.1 × 0.4
2	1	11,12	1.0	12	4.1 × 2.9 × 0.4
3	2	15	1.5	6	2.0 × 2.0 × 0.4
4	2	16	1.5	5	2.0 × 1.7 × 0.3
5	3	6	3.5	5	2.5 × 2.0 × 0.3
6	3	8	3.5	13	3.0 × 2.5 × 0.5
7	4	3,4,5	4.5	16	3.2 × 2.6 × 0.4
8	4	7	4.5	15	2.5 × 2.5 × 0.5
9	5	13	7.5	20	3.0 × 3.0 × 1.0
10	6	14	9.0	28	3.5 × 3.0 × 1.0
11	7	9	10.5	32	5.1 × 3.2 × 1.1

A histopathologic diagnosis of fibroadenoma and/or fibroadenomatous changes was confirmed from all 11 of the ultrasound-guided 8-gauge vacuum-assisted excision procedures performed.

The patient's hormonal contraception was discontinued after her third ultrasound-guided 8-gauge vacuum-assisted excision session. A resultant decrease in lesion size was noted in lesion number 9, 13, and 14 on repeat diagnostic ultrasound (Table 1) performed at a later time but prior to the time of the last three ultrasound-guided 8-gauge vacuum-assisted excision sessions (session number 5, 6, and 7) for lesion number 13, 14, and 9, respectively (Table 2). This effect appeared to be directly related to the discontinuation of her hormonal contraception (Table 1). This ultimately allowed for successful complete ultrasound-guided 8-gauge vacuum-assisted excision of the two lesions (lesion number 9 and 14) that were previously deemed as too large for consideration of attempted ultrasound-guided 8-gauge vacuum-assisted excision.

**Figure 3:**
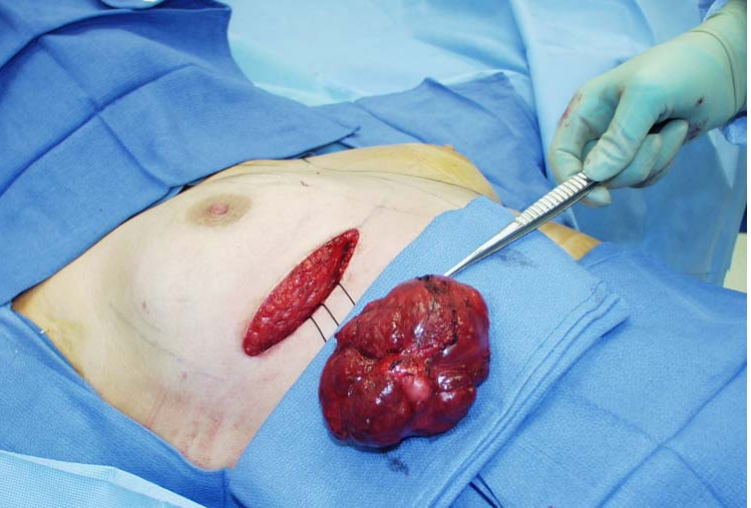
Intraoperative photograph showing removal of the giant fibroadenoma through an inframammary incision after the entire right breast parenchymal tissue plate was elevated off of the underlying right pectoralis major muscle.

Between the fourth and fifth ultrasound-guided 8-gauge vacuum-assisted excision sessions, the patient was taken to the operating room and underwent surgical excision of two right breast lesions. This consisted of a giant fibroadenoma (Figure 3) encompassing her entire central right breast (lesion number 1, Table 1) that measured 10.5 × 8.0 × 5.5 cm in size on gross pathologic evaluation, as well as an additional fibroadenoma in the 3 o'clock axis of the periareolar aspect of her right breast (lesion number 2, Table 1) that measured 1.5 × 1.2 × 0.9 cm in size on gross pathologic evaluation and which was located in close proximity along the superficial lateral aspect of the giant fibroadenoma. As is shown in Figure 3, for the surgical excision of these two right breast lesions, the patient's right breast was approached through a 10 cm inframammary incision. Through this inframammary incision, the entire right breast parenchymal tissue plate was elevated off of the underlying right pectoralis major muscle and the giant fibroadenoma was delivered out through the posterior breast tissue plate by incising the very thin rim of posteriorly compressed breast parenchyma lying just beneath the giant fibroadenoma. In this manner, the giant fibroadenoma and the second closely-adjacent smaller fibroadenoma were removed without placing any incisions upon the face of the right breast and without incising though any breast parenchyma other than the thin rim of posteriorly compressed breast parenchyma lying beneath the giant fibroadenoma. This particular surgical approach was instrumental to maximizing the cosmetic outcome.

**Figure 4:**
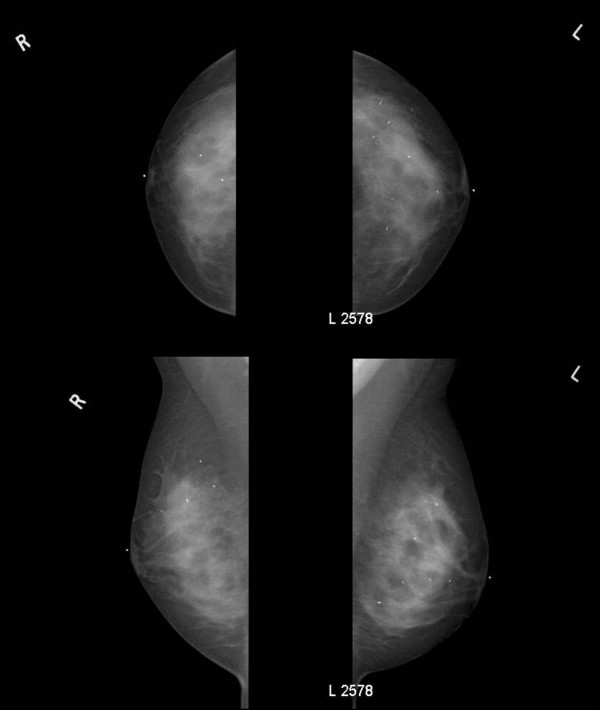
Bilateral digital mammography. Upper left panel is right cranial-caudal view. Upper right panel is left cranial-caudal view. Lower left panel is right medial-lateral-oblique view. Lower right panel is left medial-lateral-oblique view. Four individual microclips are visible within the right breast and eight individual microclips are visible within the left breast.

Approximately 18 months after the first ultrasound-guided 8-gauge vacuum-assisted excision procedure and approximately 8 month after the last ultrasound-guided 8-gauge vacuum-assisted excision procedure, the patient returned for interval follow-up and underwent bilateral digital mammography and bilateral whole breast ultrasound. Bilateral digital mammography (Figure 4) showed multiple clips within both breasts marking the areas of the 11 separate ultrasound-guided 8-gauge vacuum-assisted excision procedures that were performed during seven separate ultrasound-guided 8-gauge vacuum-assisted excision sessions. Bilateral whole breast ultrasound showed no residual ultrasound lesions at any of the sites of the 11 separate ultrasound-guided 8-gauge vacuum-assisted excision procedures and no residual ultrasound lesions at the sites of the two surgically excised right breast fibroadenomas. Likewise, bilateral whole breast ultrasound showed no new apparent ultrasound lesions elsewhere in either breast.

## Discussion

Numerous reports in the literature describe to varying degrees the use of the previously mentioned ultrasound-guided breast biopsy system for attempted removal of appropriately selected presumed benign breast lesions by both the 11-gauge platform [2-9,11-17] and the 8-gauge platform [2-10]. However, to date, there has not been a single report in the literature that addresses how this particular 8-gauge system can be used for optimally managing a patient who may present with multiple bilateral presumed benign breast lesions.

The current report represents the first account in the literature to describe the use of an ultrasound-guided 8-gauge vacuum-assisted breast biopsy system as the predominant interventional method of excision for attempting complete eradication of multiple bilateral fibroadenomas in an individual patient who presented with 16 documented breast lesions. In this particular patient, this innovative ultrasound-guided approach allowed for accomplishing complete excision of 14 separate breast lesions with virtually no aesthetic alterations to her breasts. As a result, maximal and optimal cosmetic outcome was attained and much more radical and body-altering surgical intervention (such as the bilateral nipple-sparing mastectomies and expander-to-implant reconstruction that was previously recommended by the breast surgeon at an out-of-state university medical center) was completely avoided in this particular case. Therefore, the presentation of this case nicely illustrates the contention of the present author that this ultrasound-guided 8-gauge vacuum-assisted excision technique should be considered as a viable alternative or as an adjunct to consideration of much more radical open surgical approaches when faced with the daunting task of removing multiple bilateral fibroadenomas. As such, this innovative approach is highly recommended in similar appropriately selected patients.

The discontinuation of the patient's hormonal contraception appeared to be highly effective in successfully reducing the size of two of the left breast lesions (lesion number 9 and 14) and was therefore crucial in allowing for successful complete excision of these two lesions that were previously deemed as too large for consideration of attempted excision by the ultrasound-guided 8-gauge vacuum-assisted excision technique, based on the current author's previously published size and shape criteria for predicting successful lesion excision [10]. The concept of hormonal contraception stimulating the growth of pre-existing fibroadenomas is not a new one [18,19] and this concept is further re-enforced by the findings presented within the current report. Therefore, consideration of a trial period of discontinuation of hormonal contraception can be used to attempt to convert a difficult situation in which surgical excision of one or more fibroadenomas is being contemplated to that of a more ideal situation in which attempted ultrasound-guided 8-gauge vacuum-assisted excision is possible.

Frequently, when a patient presents with a palpable event that represents a fibroadenoma, it is not uncommon to identified one or more additional lesions within the ipsilateral breast or contralateral breast on diagnostic breast ultrasound that represent other nonpalpable fibroadenomas candidates. This particular situation represents a scenario that is extremely appropriate for consideration of using an 8-gauge vacuum-assisted breast biopsy system for attempting ultrasound-guided excision of all presumed fibroadenoma candidates. Such an innovative and pro-active approach may allow for achieving complete eradication of multiple fibroadenomas candidates and avoiding later surgical excision of these lesions if their continued growth pattern were to later preclude future attempted ultrasound-guided 8-gauge vacuum-assisted excision. This approach would be particularly useful for those patients who you may suspect are inclined not to adhere to a regimented follow-up schedule for ongoing monitoring of such lesions that you may otherwise leave in place for such an ongoing monitoring strategy. Likewise, this approach would also be particularly useful for those patients on hormonal contraception in whom you are concerned about ongoing stimulation of the growth pattern of pre-existing fibroadenomas in order to prevent a situation in which a fibroadenoma which was previously amendable to attempted ultrasound-guided 8-gauge vacuum-assisted excision then becomes only removable by a surgical excision procedure. For obvious reasons, such an approach which maximizes cosmetic outcome by eliminating the necessity for creating major surgical scars on the breast is one that is of particular importance to young female patients and should be factored into the decision-making process of how to manage these patients.

As a last point of interest, and has been previously discussed by the present author [10], the ultrasound-guided 8-gauge vacuum-assisted excision technique allows for on-going, real-time, ultrasound-guided "tailoring" of the excision cavity of any given presumed benign lesion that the operator is attempting to completely excise at the time of the procedure. This technique allows the operator to remove only as much tissue as is needed to attempt complete excision of any given presumed benign lesion. This can be particularly important in smaller-breasted women in whom lesion excision volume may be crucial in determining cosmetic outcome, especially when multiple fibroadenoma candidates are encountered. This clearly represents a significant potential advantage over that of using a percutaneous en bloc excision technique for attempted complete lesion excision, since such percutaneous en bloc techniques generally afford only a single attempt at tissue acquisition and may have a greater theoretical risk for requiring an additional diagnostic procedure for complete lesion excision when inaccurate device positioning results in incomplete lesion removal in the intended region of initial en bloc tissue acquisition.

## Conclusion

This report describes, for the first time, an innovative approach of utilizing an ultrasound-guided vacuum-assisted excision technique with the previously mentioned 8-gauge breast biopsy system [2-10] for assisting in achieving complete eradication of multiple bilateral fibroadenomas in a patient who presented with 16 documented breast lesions. Successful complete excision of 14 separate lesions was accomplished with the previously mentioned 8-gauge breast biopsy system [2-10]. Discontinuation of the patient's hormonal contraception was crucial in allowing for successful complete excision of two lesions that were previously deemed as too large for consideration of attempted ultrasound-guided 8-gauge vacuum-assisted excision. As such, this innovative approach is highly recommended in similar appropriately selected patients.

## Competing interests

The author(s) declare that they have no competing interests.

## Authors' contributions

**SPP **was the surgeon who performed each of the ultrasound-guided 8-gauge vacuum-assisted excision procedures and the surgical excision procedure. He designed the current study and collected all the data. He organized, wrote, and edited all aspects of this manuscript. He approved the final version of this manuscript.
